# Maternal smoking during pregnancy and early childhood dental caries in children: a systematic review and meta-analysis

**DOI:** 10.1186/s12903-024-04548-4

**Published:** 2024-07-12

**Authors:** Dorsa Samani, SeyedMehdi Ziaei, Farhan Musaie, Hooman Mokhtari, Rubina Valipour, Mahsa Etemadi, Narin Gharehdaghi, Seyede Fateme Rezaei, Soheil Raji, Tara Fazel, Alireza Mokhtari Sakhvidi, Niloofar Deravi

**Affiliations:** 1 Tabriz University of Medical Sciences, Tabriz, Iran; 2 Hamadan University of Medical Sciences, Hamadan, Iran; 3 Dental Branch, Islamic Azad University, Tehran, Iran; 4 Faculty of Dentistry, Babol University of Medical Sciences, Babol, Iran; 5 Faculty of Dentistry, Semmelweis University, Budapest, Hungary; 6 Department of Periodontology, School of Dentistry, Tehran University of Medical Sciences, Tehran, Iran; 7 Faculty of Dentistry, Istanbul Okan University, Tuzla Campus Tuzla, Istanbul, 34959 Turkey; 8 School of Dentistry, Mazandaran University of medical sciences, Dentist, Sari, Iran; 9 Faculty of Dentistry, Van Yüzüncü Yıl University, Van, Türkiye Turkey; 10 Student Research Committee, School of International Campus, Guilan University of Medical Sciences, Rasht, Iran; 11 Dental Faculty, Tehran Medical Sciences, Islamic Azad University, Tehran, Iran; 12 Student Research Committee, School of medicine, Shahid Beheshti University of Medical Sciences, Tehran, Iran; 13 SBUMS, Arabi Ave, Daneshjoo Blvd, Velenjak, Tehran 19839-63113 Iran

**Keywords:** Maternal smoking, Pregnancy, Dental caries, Children, Systematic review, Meta-analysis

## Abstract

**Background:**

Early childhood dental caries, or ECC, is a significant global oral health concern associated with various adverse outcomes. This systematic review and meta-analysis aimed to investigate the potential link between maternal smoking during pregnancy and the occurrence of dental caries in children.

**Method:**

Through a comprehensive search of PubMed, Scopus, and Google Scholar databases for studies examining the correlation between maternal smoking during pregnancy and childhood caries, we identified 609 relevant articles up to October 2023. Studies were selected, and data extraction was based on the pre-established eligibility criteria and items. Meta-analysis was executed utilizing Comprehensive Meta-analysis (CMA) with a random effects model, ensuring a robust synthesis of the gathered evidence.

**Result:**

7 cohorts and five cross-sectional studies, totaling 12 studies, were included in our analysis. The combined results from the studies revealed a significant association between maternal smoking during pregnancy and an increased risk of dental caries in children (OR = 1.78, 95% CI = 1.55–2.05, I2 = 68.53). Sensitivity analyses confirmed the reliability of our results. However, there were indications of publication bias, as suggested by the funnel plot and Egger’s test (*P* = 0.011) concerning the connection between prenatal smoking and childhood caries.

**Conclusion:**

This review underscores the association between maternal smoking during pregnancy and childhood dental caries. Nevertheless, confounding variables influence this link, necessitating more large-scale, longitudinal studies with adjusted factors. Additional randomized control trials are needed to validate these findings due to the observed heterogeneity. Future research should investigate the precise reasons behind this association. It is essential to raise awareness among pregnant women about the risks of smoking through educational programs.

## Introduction

Early childhood dental caries is linked to various detrimental outcomes, including discomfort, tooth loss, hindered growth, reduced weight gain, diminished quality of life, impaired academic performance, and increased risk of future dental caries [1, 2]. Behavioral and lifestyle factors, such as oral hygiene and food, are well-established risk factors in the pathogenesis of dental decay [3].

Enhancing the availability of preventive dental care for young children is crucial to addressing their dental needs. Prioritizing children at high risk of tooth decay can effectively fulfill this requirement [4]. Smoking while pregnant can have detrimental effects on the oral health of the child, regardless of the specific trimester when the smoking took place [5, 6].

A meta-analysis study in 2021 encompassed a combined total of 11 studies. Out of these, six studies were cross-sectional, while three were longitudinal. Finally, the meta-analysis included the findings from these selected studies. The pooled estimates demonstrated that maternal smoking during pregnancy was significantly associated with dental decay in children both in cross-sectional studies (OR = 1.57, 95% CI = 1.47–1.67) and longitudinal studies (RR = 1.26, 95% CI = 1.07–1.48) [7].

After this meta-analysis, a 2023 cross-sectional study found that prenatal smoking was associated with an increased risk of severe caries in early childhood [8]. Also, in a longitudinal study conducted in 2023, previous maternal pregnancies and maternal smoking were significantly associated with childhood caries [9].

Because new articles have been published, this meta-analysis is an update about maternal smoking during pregnancy and dental caries in children.

## Method

This systematic review and meta-analysis aim to investigate the impact of Maternal smoking during pregnancy on dental caries in children. The exposure in our study is maternal smoking during pregnancy, under study population is pregnant women and their children, and outcome is dental caries in children. Our research methodology is in accordance with the Preferred Reporting Items for Systematic Review and Meta-analysis (PRISMA) 2020 edition. The protocol of this article was registered on The Open Science Framework.

### Search strategies

An advanced systematic literature search was conducted up to November, 2023, to collect appropriate articles from PubMed, Scopus, and Google Scholar databases using MeSH terms and three primary keywords, including maternal smoking, pregnancy, and dental caries (Table 1). No other search filter about the date, publication type, or language was applied to narrow the results. The query was minorly adjusted and modified to improve searching regarding the format of each database. Further screening of the reference lists of relevant systematic reviews was carried out, and appropriate studies were included to prevent the neglect of valuable papers. The duplicate records were found and excluded as well. Two reviewers performed the above steps, and any discrepancy was solved after consultation between reviewers.

**Table 1:** Search strategy

Search engine	Search strategy	Additional filters	Search Results
PubMed	((Maternal[Title/Abstract]) OR (Mothers[Title/Abstract])) OR (Pregnancy[Title/Abstract]) OR (Pregnant Women[Title/Abstract])) AND ((Smoke[Title/Abstract]) OR (Smoking[Title/Abstract]) OR (Nicotine[Title/Abstract]) OR (Cigarette Smoking[Title/Abstract])) AND ((Dental Caries[Title/Abstract]) OR (Dentistry[Title/Abstract]) OR (Dental[Title/Abstract]) OR (Tooth[Title/Abstract]) OR (Dental Health Services[Title/Abstract]))	**English** **November** **15th** ^**2023**^	**238**
Scopus	TITLE-ABS (maternal) OR TITLE-ABS ( mothers ) OR TITLE-ABS ( pregnancy ) OR TITLE-ABS ( pregnant AND women ) AND TITLE-ABS ( smoke ) OR TITLE-ABS ( smoking ) OR TITLE-ABS ( nicotine ) OR TITLE-ABS ( cigarette AND smoking ) AND TITLE-ABS ( dental AND caries ) OR TITLE-ABS ( dentistry ) OR TITLE-ABS ( dental ) OR TITLE-ABS ( tooth ) OR TITLE-ABS ( dental AND health AND services )	**English** **November** **15th** ^**2023**^	**313**
Google Scholar	(Pregnancy OR prenatal) AND (Smoking OR tobacco use OR cigarette smoking) AND (caries OR tooth decay OR dental caries OR tooth OR teeth OR dental OR oral health OR Dentistry)	**English** **November** **15th** ^**2023**^	**97**
Manual Search	-	**English** **November** **15th** ^**2023**^	**6**

Then, the articles’ titles, abstracts, and full texts were reviewed. Relevant studies eligible for our inclusion criteria were included. Two reviewers performed the whole procedure, and any inconsistency was resolved through consensus discussion.

### Inclusion and exclusion criteria

All the observational studies discussing maternal smoking and children’s dental care were included. Any subject irrelevant to the mentioned topic was dismissed; Studies in which their exposure was maternal smoking during infancy or childhood (not pregnancy), or their outcome was dental caries in adolescents or adults (not children), are examples of studies with irrelevant subjects. Our exclusion criteria were non-English language, in vivo studies, review articles, letters to the editor, and abstracts. In addition, all papers with insufficient data were excluded.

### Data extraction and study quality assessment

Two independent researchers performed the data extraction process. The data extraction checklist included authors, year of publication, study design, number of participants, age, and dental caries. Two independent authors conducted the quality assessment of our article according to JBI’s checklist.

### Statistical analysis

Meta-analysis was performed using CMA (Comprehensive Meta-analysis) with a random effects model. The I2 statistic was applied to calculate heterogeneity (I2 more than 75% was considered high heterogeneity, more than 50% moderate, and 25% low heterogeneity). The odds ratio for the overall effect of smoking in pregnancy on children’s caries from each study was used, which was the primary outcome reported in the included literature. The ratio was calculated from the available data, wherever feasible. The funnel plot method was used to assess publication bias. We conducted subgroup analyses based on region, children’s age, sample size, and the caries assessment technique to determine the factors affecting the pooled results. Sensitivity analyses were performed to confirm the robustness of our findings.

## Result

### Study selection

**Fig. 1:**
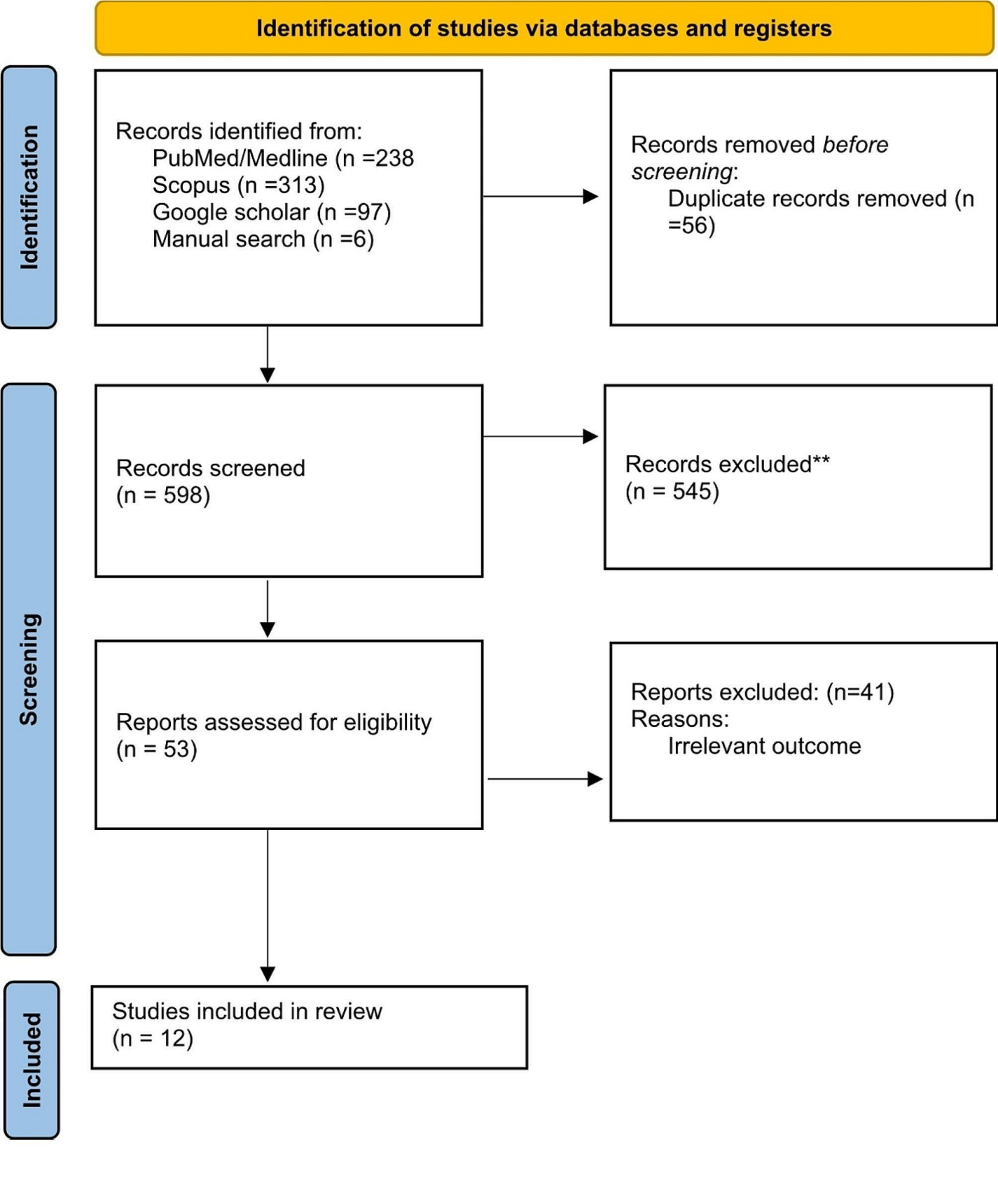
Prisma flow diagram of the study

Searching databases, including PubMed, Scopus, Google Scholar, and Manual, yielded 654 records. Fifty six studies were recognized as duplicates automatically. The remaining papers underwent primary screening of title and abstract, which removed 545 irrelevant articles. Secondary screening was performed using the full text of 53 articles. Finally, twelve studies were included in this systematic review and meta-analysis (Fig. 1).

### Study characteristics

We included twelve studies with a total of 90,405 participants, all published between 2007 and 2023 in English. The study design was cross-sectional [8, 10–13] and cohort [5, 14–19]. These studies were conducted in various countries, including England [5], Japan [11, 16–18], Australia [19], Poland [8, 12], The U.S.A [13], Sweden [14], Italy [10], and Singapore [15]. The ages of children in eight [5, 11–14, 17–19] and four [8, 10, 15, 16] studies were ≥ and < 3 years (Table 2 ).

**Table 2:** Baseline characteristic of included studies

Author (Year) [Ref]	Country	Study design	Sample	dental examination
Akinkugbe et al. (2021) [5]	England	Cohort	1,429	Clinical oral examinations
Tanaka et al. (2015) [18]	Japan	Cohort	6,412	visual examination
Claudia et al. (2016) [19]	Australia	cohort	1,687	Parent-reported dental caries
Hanioka et al. (2008) [11]	Japan	cross-sectional	6,412	visual examination
Borowska-Strugińska et al. (2016) [12]	Poland	cross-sectional	1,131	visual/tactile examination
Lida et al. (2007) [13]	America	cross-sectional	1,576	visual/tactile examination
Julihn et al. (2018) [14]	Sweden	Cohort	65,259	Clinical and radiographic examinations
Majorana et al. (2014) [10]	Italy	cross-sectional	2,395	visual/tactile examination
Sobiech et al. (2023) [8]	Poland	cross-sectional	496	Clinical oral examinations
Kalhan et al. (2020) [15]	Singapore	Cohort	721	visual/tactile examination
Nakayama et al. (2022) [16]	Japan	cohort	872	visual examination
Tanaka et al. (2009) [17]	Japan	Cohort	2,015	visual examination

### Meta-analysis and publication bias

**Fig. 2:**
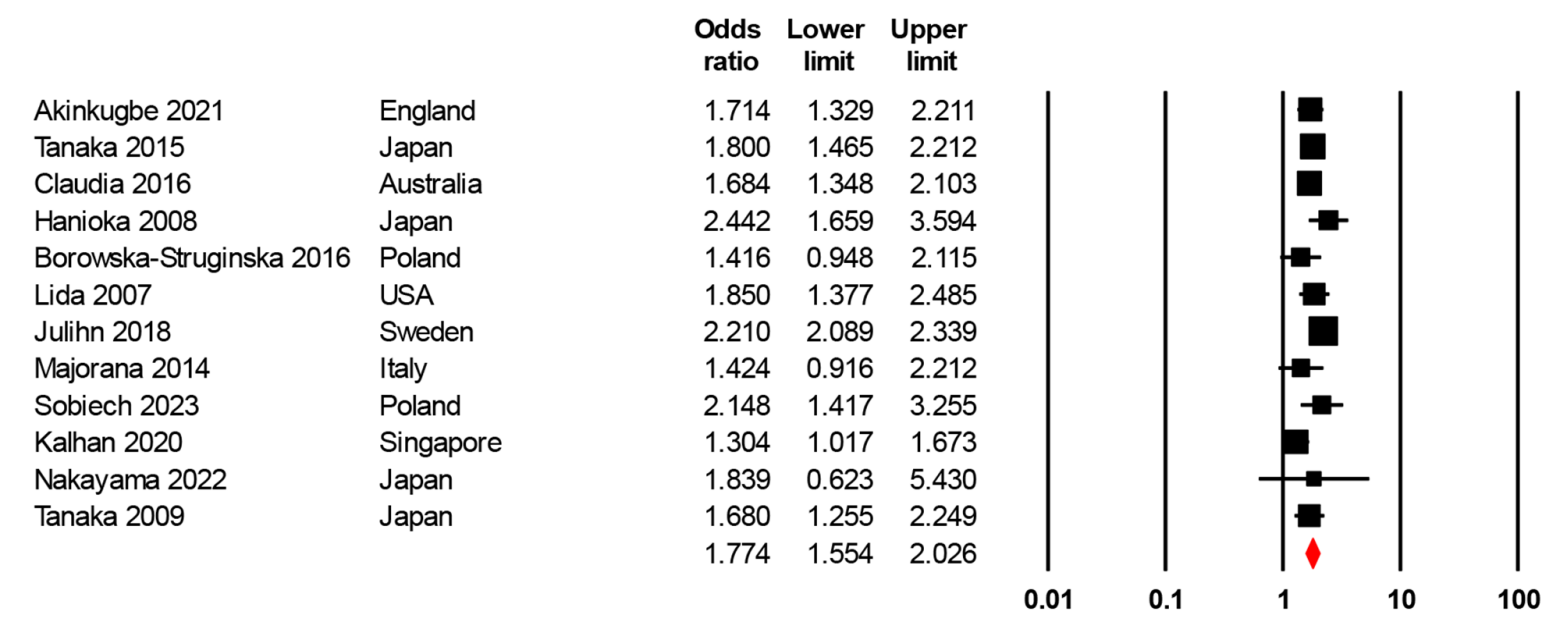
Forest plot of maternal smoking during pregnancy and dental caries in children

The pooled result of the cross-sectional studies indicated maternal smoking during pregnancy was significantly associated with the risk of dental caries in children (OR = 1.78, 95% CI = 1.55–2.05, I2 = 68.53) (Fig. 2). The sensitivity analyses suggested that our meta-analysis results were robust (Table 3).

**Table 3:** The results of subgroup and sensitivity analyses

Subgroup		No. of studies	OR (95% CI)	Heterogeneity	
				I^2^	*P*
Overall		12	1.78 (1.55, 2.05)	68.53	< 0.05
Study Region	Asia	5	1.71 (1.40, 2.10)	50.23	0.09
	Australia	1	1.68 (1.35, 2.10)	0	NA
	Europe	4	1.67 (1.40, 1.99)	0	0.457
	USA	1	1.85 (1.38, 2.49)	0	NA
Children’s age	≤ 3	7	1.70 (1.50, 1.91)	39.37	0.129
	> 3	5	1.83 (1.53, 2.18)	70	0.009
Sample size	≤ 1000	3	1.63 (1.11, 2.40)	52.56	0.121
	1000 < and ≤ 3000	7	1.70 (1.48, 1.88)	0	0.893
	> 3000	2	2.18 (2.07, 2.31)	48.38	0.144
Dental examination	Clinical oral examinations	2	1.82 (1.47, 2.26)	0	0.364
	visual examination	4	1.85 (1.59, 2.16)	0	0.480
	Parent-reported dental caries	1	1.68 (1.35, 2.10)	0	NA
	visual/tactile examination	4	1.48 (1.26, 1.74)	8.22	0.352
	Clinical and radiographic examinations	1	2.21 (2.09, 2.34)	0	NA
Sensitivity Analysis	Excluded Akinkugbe et al. [5]	11	1.78 (1.54, 2.05)	NA	NA
	Excluded Tanaka et al. [18]	11	1.77 (1.53, 2.05)	NA	NA
	Excluded Claudia et al. [19]	11	1.78 (1.55, 2.06)	NA	NA
	Excluded Hanioka et al. [11]	11	1.73 (1.51, 1.99)	NA	NA
	Excluded Borowska-Strugińska et al. [12]	11	1.80 (1.58, 2.06)	NA	NA
	Excluded Lida et al. [13]	11	1.77 (1.53, 2.04)	NA	NA
	Excluded Julihn et al. [14]	11	1.70 (1.54, 1.87)	NA	NA
	Excluded Majorana et al. [10]	11	1.80 (1.57, 2.06)	NA	NA
	Excluded Sobiech et al. [8]	11	1.75 (1.52, 2.01)	NA	NA
	Excluded Kalhan et al. [15]	11	1.85 (1.64, 2.08)	NA	NA
	Excluded Nakayama et al. [16]	11	1.77 (1.55, 2.03)	NA	NA
	Excluded Tanaka et al. [17]	11	1.78 (1.55, 2.05)	NA	NA

**Fig. 3:**
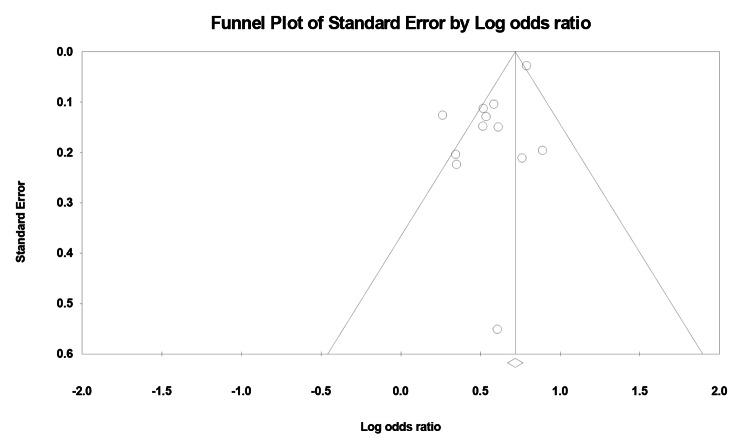
Funnel plot of maternal smoking during pregnancy and dental caries in children

The funnel plot and Egger’s test (*P* = 0.011) indicated publication bias for the correlation between prenatal smoking and childhood caries (Fig. 3).

## Discussion

This systematic review and Meta-analysis of 85,159 showed smoking during pregnancy increases the risk of tooth caries in children by 78%.

The latest systematic review and Meta-Analysis on this topic was done by Zhong et al. in 2021. They concluded that there is a significant correlation between maternal smoking during pregnancy and childhood caries. However, they reported more prospective and extensive studies on this theme are needed for verification. In our study, we included two new cohort studies by Kelhan et al. (2020) and Nakayama et al. (2022) and one new cross-sectional study by Sobiech et al. (2023) to have more valid results [7, 8, 15, 16].

The development of primary tooth germs typically commences between 7 and 10 weeks of gestation, with subsequent enamel and dentine deposition. The primary teeth’ mineralization begins at 13 weeks of gestation and concludes by the end of the first year of life. Various studies have identified potential impacts of smoking, particularly nicotine, and cotinine, on deciduous teeth, but the exact processes responsible for the adverse effects of smoking during pregnancy on the development of dental cavities in children have not been fully elucidated. However, several studies have proposed potential explanations for these effects. Some of the possible reasons are listed below [17, 18].

1. Smoking can postpone the progression of dental development [18].
2. Contrary to the previous study, another research has indicated that maternal smoking during the first trimester of pregnancy can expedite the process of tooth formation, resulting in earlier tooth eruption. Additionally, there is evidence of a correlation between exposure to secondhand smoke (SHS) and the premature eruption of primary teeth [5, 8].
3. Smoking acts as a contributing factor in the development of early childhood caries (ECC) after prenatal exposure of the developing tooth to detrimental chemical toxins. It is important to acknowledge that teeth are highly vulnerable to disruptions in mineralization caused by environmental toxins [15, 17].
4. The proliferation of cariogenic bacteria in mothers is observed to increase following nicotine consumption, leading to vertical transmission from mothers to their children. Elevated levels of cariogenic bacteria in smoking mothers, as compared to non-smoking mothers, are recognized as a significant risk factor for Early Childhood Caries (ECC) [8, 15].
5. Tobacco use during pregnancy has been found to impede the normal morphological development of enamel, leading to the formation of defects that elevate the susceptibility to dental caries. The presence of irregularities and retentive grooves on the tooth surfaces may significantly contribute to the colonization and accumulation of cariogenic bacteria by promoting the adherence of bacterial plaque [5, 7, 17, 19].
6. Nicotine facilitates Streptococcus mutans’ adherence to dental surfaces, which leads to increased biofilm formation, heightened metabolic activity within the biofilm, and elevated bacterial counts [8, 18].
7. Nicotine can influence cell specialization, proliferation, and maturation [17, 18].
8. Smoking can influence the inductive capabilities of dental papillae, the enamel organ, and the subsequent odontogenesis [17].
9. Nicotine has been found to impede the mineralization process of tooth buds by inhibiting matrix synthesis and mineralization in ameloblasts and odontoblasts. This interference can lead to reduced mineralization of odontoblast cells within human dental pulp cells and disrupt enamel mineralization, potentially resulting in hypoplasia or hypomineralization of teeth. Consequently, such teeth may become more vulnerable to dental caries. Additionally, researchers suggest that a high-sugar diet should facilitate the progression of enamel hypoplasia to early childhood caries (ECC) [8, 14, 17–19]. Smoking can Lower vitamin D levels in the bloodstream, which can impact the mineralization of teeth, potentially leading to adverse effects during the mineralization period. Researchers have shown a negative correlation between serum cotinine levels and serum vitamin D concentration. Additionally, lower maternal prenatal 25-hydroxyvitamin D levels in serum and reduced maternal vitamin D intake during pregnancy have been linked to an increased risk of dental caries in children [18, 20].
10. Smoking has been found to modify salivary composition by reducing its buffering capacity, changing its chemical composition, altering bacterial constituents, declining PH, decreasing salivary flow, and consequently facilitating the development of an environment conducive to dental caries. Additionally, enamel hypoplasia has been linked to elevated levels of cariogenic bacteria in saliva [8, 17].
11. Dental caries is a persistent bacterial infectious disease affecting the oral cavity. The potential association between exposure to tobacco smoke and caries may be linked to changes in the body’s immune responses. It is plausible that smoking could make children more susceptible to infections by suppressing or altering the immune system. Furthermore, it is understood that immunosuppression may facilitate the colonization of Streptococcus mutans. Smoking has been shown to negatively impact the functioning of immune cells, such as fetal T-helper 1 and 2 cells, and inhibit the phagocytic activity of neutrophils and monocytes by its nicotine. Also, researchers suggest that specific components in cigarette smoke may excessively stimulate the body’s response, and intensifying the phagocytic activity of salivary polymorphonuclear leukocytes is an example of this effect [8, 14, 17].
12. The colonization of Streptococcus mutans may be influenced by the impact of maternal smoking during pregnancy on the timing of primary teeth eruption and the maturation of salivary glands [11].
13. Children whose mothers smoked during pregnancy were less likely to be breastfed, which may be indicative of poor dietary and oral hygiene habits. Additionally, poor oral health awareness, habits, and behavioral factors such as the frequency of sugar-containing snack consumption and daily tooth brushing by parents were significantly linked to parental smoking status and may contribute to the development of tooth decay in children [8, 10, 11, 13, 18].

Studies that have been used in our meta-analysis have shown a positive correlation between smoking during pregnancy and increased dental caries in children and supported the results of our research.

In a 2023 cross-sectional study, Sobiech et al. examined 496 children aged 12–36 months in Poland to explore the correlation between maternal smoking, socio-behavioral factors, and dental caries in toddlers. The study findings indicated that prenatal smoking is linked to a higher likelihood of severe early childhood caries (S-ECC) [8].

In a study conducted in 2022, Nakayama and colleagues examined 872 children aged 18 to 23 months in Hokkaido, Japan, with subsequent follow-up at three years of age to explore the risk factors for Early Childhood Caries. Their findings indicated a significant association between maternal smoking during pregnancy and an increase in DMFS [16].

In a 2021 cohort study conducted by Akinkugbe et al., 1429 mother-offspring participants were examined to assess the potential impact of prenatal smoking on the risk of early childhood caries (ECC) in England, specifically considering the trimester during which smoking occurred. The study findings indicated that smoking during pregnancy may have detrimental effects on the oral health of offspring, regardless of the trimester in which the smoking took place. Furthermore, it was observed that smoking during the third trimester of pregnancy was associated with a higher risk of dental caries in offspring compared to smoking during the first or second trimester [5].

In a 2020 cohort study by Kalhan and colleagues, 1,176 healthy pregnant women in Singapore were examined to assess caries risk prediction. The study findings indicated that prenatal tobacco smoke exposure was identified as a significant risk factor for early childhood caries (ECC) development in children between the ages of 2 and 3 years [15].

In a 2018 cross-sectional study, Julihn et al. examined 65,259 children aged 3 and 7 in Sweden to explore the association between maternal health during pregnancy and child health. The findings indicated that maternal obesity and smoking in early pregnancy were significant indicators of dental caries experience in preschool-aged children [14].

In a 2015 cross-sectional study, Tanaka and colleagues examined 6412 children to explore the correlation between prenatal exposure to maternal smoking and postnatal exposure to household smoking with dental caries in 3-year-old Japanese children. Their findings indicated that maternal smoking during the initial trimester of pregnancy was linked to a notable 40% heightened likelihood of dental caries in children. Consequently, the first trimester was identified as the period during which exposure to maternal smoking poses the most significant risk of dental caries in children [18].

In a 2014 cross-sectional study, Majorana et al. examined 2395 Italian toddlers between the ages of 24 and 30 months to assess the impact of feeding and smoking habits as combined risk factors for early childhood caries (ECC). Their findings indicated that toddlers who were fed with infant formula and exposed to smoke during pregnancy, particularly those residing in areas with lower mean housing prices per square meter, exhibited higher levels of caries severity [10].

In a 2009 cross-sectional study, Tanaka et al. examined 2015 children aged Three years in Japan to assess the impact of maternal smoking during pregnancy and postnatal household smoking on dental caries in young children. The study revealed that both maternal smoking during pregnancy and postnatal household smoking were individually linked to a higher prevalence of dental caries in young Japanese children. Furthermore, the research indicated that maternal smoking during pregnancy, even without subsequent postnatal environmental tobacco smoke (ETS) exposure at home, was independently associated with an increased prevalence of dental caries. The study’s findings are significant as they demonstrate the independent effects of in-utero exposure to maternal smoking and postnatal ETS exposure, which has been challenging to study due to the likelihood of women who smoke during pregnancy continuing to smoke after delivery [17].

In a 2007 cross-sectional study, Lida et al. examined 1576 children aged 2 to 5 in the United States to explore the relationship between infant breastfeeding and Early Childhood Caries (ECC). The study identified poverty, Mexican American ethnic background, and maternal smoking as independent risk factors for ECC. The research found that children whose mothers reported smoking during pregnancy were less likely to be breastfed. However, maternal smoking during pregnancy was also independently linked to higher rates of ECC and increased caries in multivariable analyses that controlled for breastfeeding. The data did not allow for a clear distinction between prenatal, postnatal, or combined tobacco smoke exposure as contributors to the elevated risk of ECC, as women who smoked during pregnancy were likely to continue smoking postnatally. Additionally, the study suggested that maternal smoking during pregnancy might serve as an indicator of a mother’s unhealthy dietary choices and oral hygiene practices [13]. 

In a 2007 cross-sectional study, Hanioka et al. examined 711 36-month-old children in Japan to investigate the correlation between parental smoking status and dental caries in 3-year-old children. The findings revealed a link between early childhood caries (ECC) and parental smoking, with a weaker association observed for paternal smoking compared to maternal smoking. The study demonstrated an association between dental caries prevalence in 3-year-old children and parental smoking, with a weaker association observed for paternal smoking. However, the potential influence of smoking by other household members cannot be discounted [11].

One of the included studies reported a weak association between prenatal smoking and dental caries in children.

In a 2016 cohort study, Claudia et al. examined 1,687 Indigenous Australian children aged 5 or younger in Australia to explore the correlation between maternal smoking during pregnancy and parental-reported incidence of dental caries. The study indicates a tenuous link between maternal smoking during pregnancy and the prevalence of parentally-reported dental caries in Indigenous Australian children. Furthermore, the research demonstrates that sugar consumption and low maternal educational attainment were the most influential factors associated with the onset of parent-reported dental caries in this demographic. In summary, the study identified a modest positive association between maternal smoking during pregnancy and the prevalence of parent-reported dental decay in Australian Indigenous children [19].

Certain constraints must be acknowledged in the current systematic review and meta-analysis.

1. The majority of the articles included in our meta-analysis utilized cross-sectional study designs, which precluded the establishment of a causal relationship between maternal smoking during pregnancy and childhood caries. Although our analysis incorporated four cohort studies by Nakayama et al., Claudia et al., Kelhan et al., and Akinkugbe et al. that yielded consistent findings, additional prospective longitudinal research is warranted to substantiate our hypothesis [5, 7, 15, 16, 18, 19].
2. Numerous potential confounding variables may have an impact on the outcomes. It is hypothesized that the association between maternal smoking during pregnancy, exposure to secondhand smoke, and childhood caries may be attributed to the intersection of socioeconomic, educational, and behavioral factors. It is plausible that our findings are still confounded by other potentially significant aspects of children’s experiences that could impact their oral health, such as the frequency of nighttime feedings and patterns of dental visits. Subsequent research in this area should be meticulously designed to mitigate the potential influence of confounding factors on the results [7, 8, 17].
3. We did not assess the relationships between different levels of caries severity with prenatal smoking due to insufficient data availability [7].
4. Studies included in the analysis utilized self-reported data to evaluate exposure. However, this approach has limitations, including the potential for recall bias due to retrospective reporting and its impact on the final results of the meta-analysis and diminishing the observed association between maternal smoking exposure and dental caries. For instance, smoking during pregnancy may be underreported due to the known adverse effects on fetal development, social desirability, and incomplete reporting. Nevertheless, it is essential to recognize that pregnant women are generally aware of the need to limit exposure to harmful substances such as tobacco smoke, which may enhance the accuracy of their recall. Despite these limitations, parental reporting is a reliable method when clinical data collection is not feasible due to logistical and cost constraints [5, 7, 10, 17–19].
5. The available data does not allow for a clear distinction to be made regarding whether the elevated risk of childhood caries is linked to prenatal, postnatal, or both prenatal and postnatal exposure to tobacco smoke. This is due to the likelihood that women who smoke during pregnancy are inclined to continue smoking after childbirth. Additionally, it is plausible that maternal smoking during pregnancy may serve as an indicator of a mother’s unhealthy dietary choices and oral hygiene practices. Only one of the included studies by Tanaka et al. in 2009 succeeded in distinguishing prenatal and postnatal smoking effects [7, 13].
6. Different studies utilized Various approaches for assessing dental caries, which could affect our study results [18].
7. One variable that may have impacted the outcomes of the studies included in the analysis is the duration of the smoking habit, which has not been investigated in the included studies. It is hypothesized that the children of women who have smoked for a more extended period may be at a higher risk of childhood caries compared to those with a shorter history of smoking.

Another variable is the trimester of pregnancy in which smoking occurs, which is only considered in 3 studies with contradicted results. Akinkugbe et al. have reported the third trimester of pregnancy as the most associated trimester with caries in children, unlike Tanaka and Julihn studies, which have reported, respectively, first trimester and early pregnancy as periods with higher risks [5, 7, 14, 18, 19].

The authors of the current systematic review suggest that in addition to providing treatment for early childhood caries (ECC) in children, it is essential for oral healthcare professionals to educate parents or guardians about the harmful effects of smoking on health, particularly during pregnancy. Pregnant women should become more educated about the dangers of smoking during pregnancy through different programs. Further research is necessary to validate these findings and to comprehend the mechanisms underlying the observed correlation between prenatal and postnatal exposure to environmental tobacco smoke (ETS) and dental caries in young children [7, 17].

## Conclusion

In summary, the current systematic review and meta-analysis have demonstrated a link between maternal smoking during pregnancy and dental caries in children. However, this association is influenced by various confounding factors, which makes the need for more longitudinal and large-scale population studies with adjusted confounding factors, necessary. Also, Due to the high heterogeneity of our study, further randomized control trials are still required to support our results.

Besides, the exact reason behind the association between smoking during pregnancy and caries in children should be determined in future studies.

## Data Availability

The datasets generated and analyzed during the current study are not publicly available but are available from the corresponding author on reasonable request.
